# Magnitude and its associated factors of neonatal jaundice among neonates admitted to the neonatal intensive care unit of Dessie Town public hospitals, Amhara region, Ethiopia, 2020: a multicenter cross-sectional study

**DOI:** 10.3389/fped.2024.1288604

**Published:** 2024-01-26

**Authors:** Mohammed Tessema, Hussen Mekonnen, Tsion Alemu, Yohannes Godie, Wegayehu Zeneb Teklehaimanot, Leweyehu Alemaw Mengstie

**Affiliations:** ^1^ College of Health Science, Debre Berhan University, Debre Birhan, Ethiopia; ^2^ School of Public Health, Addis Ababa University, Addis Ababa, Ethiopia; ^3^ College of Health Science, Debre Markos University, Debre Markos, Ethiopia

**Keywords:** neonatal jaundice, magnitude, determinants, Dessie Town, Northeast Ethiopia

## Abstract

**Background:**

Neonatal jaundice is a prevalent illness affecting approximately 60%–80% of newborns. In severe cases, it can result in severe neurological distress. Approximately 1.1 million neonates are affected annually on a global scale, with the vast majority living in sub-Saharan Africa and southern Asia. It is common in newborns in the first week of life. This study aims to assess the magnitude and determinants of jaundice in newborns admitted to the neonatal intensive care unit (NICU) of public hospitals in the city of Dessie in northern Ethiopia.

**Methods:**

An institutional cross-sectional study was conducted at the NICU at Dessie town public hospitals from 30 March to 30 April 2020. A systematic random sampling procedure was used to select the study participants. Data were collected through face-to-face interviews with mothers using a structured questionnaire and by reviewing neonatal medical records using a checklist. Binary logistic regression analysis was used to identify the determinants of neonatal jaundice. A significance level of less than 0.05 was used to declare the statistical significance in the final model.

**Results:**

A total of 218 neonates with their mothers were included in the study. The prevalence rate of neonatal jaundice was found to be 28.4%. The major associated factors for neonatal jaundice were sepsis [adjusted odds ratio (AOR): 10.13, 95% confidence interval (CI) = 2.36, 43.56], preterm < 37 weeks (AOR: 6.03, 95% CI = 1.41, 25.79) low APGAR score < 7 (AOR: 7.34, 95% CI = 1.34, 39.65), ABO incompatibility (AOR: 24.55 95% CI = 1.58, 68.83), prolonged labor (AOR 9.03, 95% CI = 1.67, 48.33), and Rh incompatibility (AOR = 30.40, 95% CI = 2.01, 66.20).

**Conclusion:**

The magnitude of neonatal jaundice among neonates was determined to be high. Therefore, both maternal and neonatal factors contributed significantly to the management of neonatal jaundice and also influenced the use of phototherapy treatment. Proper consideration of these factors is crucial for the prevention and treatment of neonatal jaundice.

## Introduction

Neonatal jaundice is a prevalent condition affecting 60% of term and 80% of preterm newborns to a variable degree worldwide (1). If severe jaundice develops, it can lead to acute bilirubin encephalopathy or kernicterus with a significant risk of neonatal mortality and long-term neurodevelopment squeals such as cerebral palsy, sensor neural hearing loss, intellectual difficulties, or gross developmental delays (2).

Neonatal jaundice is characterized by a yellowish discoloration of the mucous membranes, skin, and sclera due to the accumulation of unconjugated, non-polar, lipid-soluble bilirubin pigment in the skin (3). Neonatal jaundice is most common during the first few weeks of neonatal age. It is a significant concern that requires medical attention. Approximately 10% of breastfed babies continue to exhibit jaundice at 1 month of age. This illness is prevalent worldwide, with approximately 75% of hospital readmissions being attributed to it (4).

Neonatal jaundice is particularly important due to the strong association between elevated unconjugated bilirubin levels and neurotoxic effects, which can lead to long-term complications such as cerebral palsy, and hearing impairment (5). Neonatal jaundice occurs when there is an excess release of hemoglobin from the breakdown of red cells due to high levels of hemoglobin at birth, as well as due to the reduced life span of newborn red blood cells (70–80 days compared with 90–120 days in adults) and the reduced hepatic metabolism of bilirubin due to the immature liver. The majority of neonatal jaundice is a natural transition that typically resolves within the first week of life as the liver function matures (6).

Early diagnosis of high-risk infants with severe neonatal jaundice plays an important role in the timely and accurate prevention of the condition in the first 14 days after birth (7). Neonatal jaundice may have serious side effects on the health of infants; it is important to take into consideration its associated factors in newborns. Kernicterus is one of the most critical and, at times, most dangerous complications of the disease (8). Neonatal jaundice is considered one of the most dangerous signs of neonatal illness, as recognized by the World Health Organization (9). Annually, approximately 1.1 million infants worldwide experience neonatal jaundice, either with or without bilirubin encephalopathy. Of those newborns, 481,000 were born at full term. Among these, 114,000 died annually, while over 63,000 managed to survive with a moderate or severe disability. The vast majority of affected neonates reside in sub-Saharan Africa, including Ethiopia, accounting for 75% of the overall affected population (10). According to a recent review, sub-Saharan Africa and South Asia are the primary regions responsible for an estimated 1.1 million babies who would develop severe hyperbilirubinemia worldwide annually. Early identification of infants who are at risk of developing severe hyperbilirubinemia is therefore crucial for this potentially devastating condition (10). A study conducted in Calabar, South-South Nigeria, revealed that neonatal jaundice is a prevalent issue among infants admitted to the neonatal intensive care unit (NICU). The study identified the major factors associated with neonatal jaundice, including infection, i.e., septicemia (71.4%), ABO incompatibility (19.1%), glucose-6-phosphate dehydrogenase deficiency (9.5%), and gestational age <37weeks (11).

In a 2015 study conducted by Bahbah et al. (12) in Nigeria, it was found that neonatal jaundice had a strong correlation with premature neonates and developed earlier after birth in premature babies compared with full-term babies. The average gestational age of preterm neonates in the study was 32.78–34.82 weeks, and jaundice started at 1.88–5.52 days after birth. However, among full-term babies, the average gestational age was 37.28–39.12 weeks, and jaundice developed 3.36–6.44 days after birth. A retrospective study conducted in Ethiopia on the magnitude and associated factors of neonatal jaundice found that 44.9% of the neonates developed jaundice, with 89 (25%) of males and 71 (19.9%) of females affected. In this study, ABO incompatibility 57 (35.6%), sepsis 30 (18.8%), idiopathic cause 22 (13.8%), breastfeeding jaundice 16 (10%), and Rh-incompatibility14 (8.8%) were significantly associated with neonatal jaundice (13). A study conducted in Pakistan indicated that neonatal jaundice is the leading cause of neonatal mortality and morbidity, an inclusion from the global child health agenda under the Million Development Goals initiative. The study conducted in Nigeria identified several factors associated with neonatal jaundice, including hemolytic disease of the newborn, preterm birth, ABO incompatibility, infection, and other associated factors. These factors were the most increasingly acknowledged as significant contributors to the increasing neonatal mortality and morbidity worldwide (14). Therefore, it is evident that neonatal jaundice is a major health problem worldwide, especially in sub-Saharan Africa. Thus, the objective of this study is to determine the magnitude and associated factors of neonatal jaundice in newborns who were admitted to the neonatal intensive care units of public hospitals in the city of Dessie.

## Methods

### Study design, setting, and period

An institution-based cross-sectional survey was conducted in the public hospitals of Dessie Town, which is located 401 km away from Addis Ababa and 480 km from Bahir Dar. The town has a total of six hospitals, consisting of four private hospitals and two public hospitals. Of those, only two hospitals provided neonatal ICU services. Therefore, this study was conducted in Dessie Comprehensive Specialized Hospital and Boro Meda Comprehensive Specialized Hospital in a neonatal intensive care unit. The study was conducted from 30 March to 30 April 2020.

### Source and study population

The study included all populations of infants and their mothers who were admitted to the NICU of these hospitals. The inclusion criteria were all infants with a postnatal age of ≤ 28 days after birth who were admitted to the Dessie Public Hospital neonatal intensive care unit with a case of jaundice during the study period.

### Exclusion criteria

Infants whose mothers were severely ill or unable to provide informed consent, cases with incomplete registration cards, and readmissions due to jaundice during the study period were excluded from the study.

### Sample size determination and procedure

**Figure 1:**
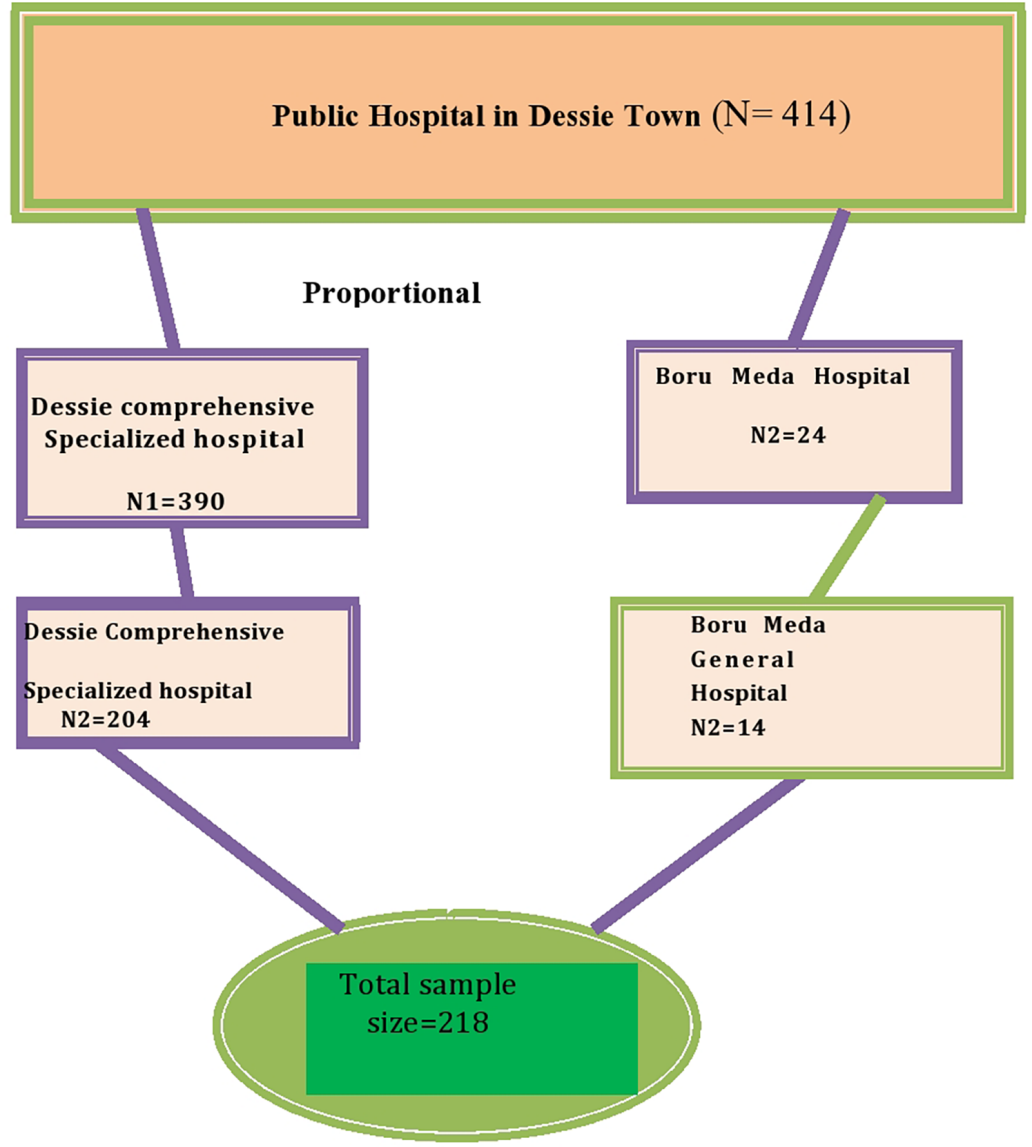
Schematic presentation of the sampling procedure for the magnitude and associated factor for neonatal jaundice among neonates admitted to the NICU in Dessie Town public hospitals, Amhara region, Ethiopia 2020.

The sample size was determined using the single population proportion formula, assuming a prevalence rate of 44.6% for neonatal jaundice in Addis Ababa in Ethiopia with a 95% confidence level and a 5% margin of error (13). The calculated sample size was 380. Since the total population was less than 10,000, a correction formula was used, and the final sample size was 218 newborns. The allocation of newborn/mother pairs surveyed from each hospital were conducted proportionally based on the expected number of case admissions in the study period, which was estimated using the number of admissions in the last 5 months at each institution. A proportional allocation formula was used for each hospital based on their admissions. Finally, the participants were selected systematically (*K* = 2) based on a sequence of admissions until the required sample size was obtained (Figure 1).

### Data collection tool and procedure

The data were collected through face-to-face interviews with mothers using a structured questionnaire and by reviewing neonatal medical records using a checklist. This method was adapted from similar studies (15–18). The questionnaire comprises four sections: (I) maternal sociodemographic factors, (II) maternal risk factors, (III) neonatal sociodemographic characteristics, and (IV) neonatal risk factors. The data were gathered by four BSc neonatal nurses with expertise in data collection, under the supervision of two experienced nurses.

### Data quality assurance

The questionnaire was initially prepared in English and translated to Amharic, then back-translated to English by two language experts. The data collectors and supervisors received a 2-day training. Moreover, a pretest was conducted on 5% (10) of the total number of participants in the Weldeya Comprehensive Hospital. Continuous supervision and daily checking were conducted by the supervisor and principal investigator.

### Data processing and analysis

The data were checked for completeness, cleaned, coded, and entered into Epi data version 3.1, then exported to SPSS version 25 for further analysis. Descriptive statistics were conducted. The Hosmer–Lemeshow goodness-of-fit test was used to determine model fitness, and the multi-collinearity test was utilized to detect any correlations among the independent variables. A *p*-value of less than 0.25 for the variable in the bivariable analysis was included in the multivariable analysis. Finally, the adjusted odds ratio with its corresponding 95% CI was computed, and a variable with a *p*-value of less than 0.05 was considered to be significantly associated with neonatal jaundice.

### Operational definitions

#### Neonatal jaundice

The results from elevated levels of total serum bilirubin and clinically manifests as a yellowish discoloration of the skin, sclera, and mucous membrane (WHO).

#### Preterm

A newborn whose gestational age is less than 37 weeks.

#### Low birth weight

The neonatal birth weight is less than 2,500 g.

#### Neonate

A neonate from birth to 28 days of the age of life.

#### Normal birth weight

The neonate weight is greater than 2,500 g and less than 4,000 g.

#### Breastfeeding jaundice

Occurs in the first few 2–3 days of life and was related to decreased breast milk intake and decreased frequency of feeding as well as a history of formula feeding that may indicate the occurrence of breastfeeding jaundice.

#### Breast milk jaundice

Late-onset jaundice occurring after the 4th–7th day of life, which is caused by increased reabsorption of unconjugated bilirubin.

## Results

### Sociodemographic characteristics of the study participants

The study included a total of 218 pairs of newborn babies and mothers, constituting a response rate of 100%. The mean age of mothers was 28.68 years (SD = 5.3), and more than half of the respondents (60.8%) were found within the age group of 20–35. Regarding maternal educational status, 32 (14.8%) of the respondents were unable to read and write, and more than half of the mothers were housewives. The majority of the respondents (188, 86.2%) were married. Nearly half of the respondents (120, 54.8%) live in urban areas. Regarding the mother's occupation, the majority (131, 59.8%) of the respondents were housewives.

### Maternal factors for neonatal jaundice

More than half (146, 67.0%) of the respondents were multipara. Regarding the mode of delivery, 202 (92.2%) deliveries were accomplished through spontaneous vaginal birth. A total of 206 (94.5%) mothers had ANC follow-up (Table 1).

**Table 1:** Maternal factor for neonatal jaundice in Dessie Town public hospitals, Amhara region, Ethiopia, 2020.

Variable (*N* = 218)	Category	Frequency	Percent
Parity	Primipara	72	33.3
	Multipara	146	67.0
Maternal blood group and Rh-factor	A	57	26
	B	70	32
	AB	29	13.8
	O	53	24.2
	Unknown	9	4
Chronic medical illness	Yes	12	5.5
	No	206	94.5
Mode of delivery	SVD	202	92.2
	C/S (C-section)	11	5.1
	Instrumental	5	3.7
Place of delivery	Home	6	2.8
	Health center	94	43.2
	Hospital	118	54
Timing of delivery	Day	43	19.7
	Night	175	80.3
Substance during pregnancy	Yes	26	11.9
	No	192	88.1
Types of substance abuse	Alcohol-taking	15	57.7
	Herbal medication	3	11.5
	Chat chewing	8	31.8
History prolonged PROM	Yes	23	10.6
	No	195	89.3
Infection during pregnancy	Yes	24	11.0
	No	194	89.0
ANC follow-up	Yes	206	94.5
	No	12	5.5
Prolonged labor	Yes	21	9.6
	No	198	90.4
Oxytocin during labor	Yes	96	44
	No	122	55
Family/sibling history of jaundice	Yes	26	11.9
	No	192	88.1

### Neonatal factor for jaundice

Among the respondents, 110 (50.5%) were male infants. Among those neonates, the majority of the age group was found between 0 and 7 days, amounting to a total of 164 (75.2%) individuals. Regarding birth weight, the majority of neonates (68.8%) had a low birth weight, and 146 (66.9%) neonates were being breastfed (Table 2).

**Table 2:** Neonatal factors for neonatal jaundice in Dessie Town public hospitals, Amhara region, Ethiopia, 2020.

Variable (*N* = 218)	Category	Frequency	Percent
Neonatal sex	Male	110	50.5
	Female	108	49.5
Neonatal age	0–7 days	164	75.2
	8–28 days	54	24.8
Birth weight	Less than 2.5–4 kg	150	68.8
	More than 2.5 kg	68	31.2
Gestational age	Less than 37 weeks	133	61
	More than 37 weeks	85	39
APGAR score	Less than 7	108	49.5
	More than 7	110	50.5
Blood group and Rh-factor	A	48	21.9
	B	92	42
	AB	27	12.8
	O	51	23.3
Neonatal sepsis	Yes	55	25.3
	No	163	74.7
Did have RH incompatibility?	Yes	12	5.5
	No	206	94.5
ABO incompatibility?	Yes	19	8.7
	No	207	91.3
Birth trauma during labor	Yes	31	14.2
	No	187	86.8
Birth asphyxia	Yes	12	5.5
	No	206	94.5
Breastfeeding method	Breastfeeding	146	66.9
	Formula feeding	25	11.5
	Mixed feeding	26	11.9
	Maintenance	21	9.7

In this study 28 (45.4%) neonates developed neonatal jaundice between 1 to 7 days of age. There were 19 (30.6%) cases in neonates less than 1 day old, while 11 (17.5%) cases occurred between days 8 and 14, and four (6.5%) cases occurred after 14 days of age.

### Proportion of neonatal jaundice

**Figure 2:**
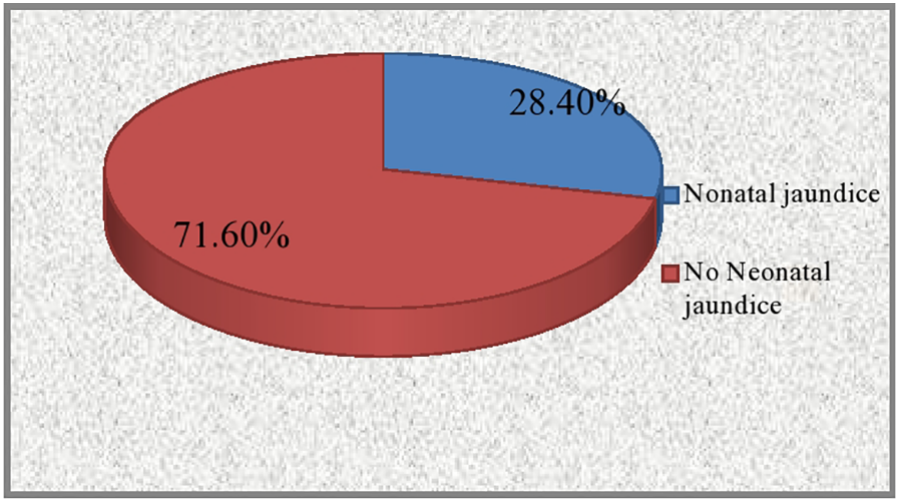
The prevalence of neonatal jaundice among neonates admitted to the NICU in Dessie Town public hospitals, Amhara region, Ethiopia 2020.

The proportion of neonatal jaundice among neonates admitted to the neonatal intensive care unit was 28.4% (62 cases) (Figure 2).

Among these, 32 (14.7%) cases were pathological jaundice, and the remaining 30 (13.7) cases were physiological jaundice. Phototherapy was administered to all jaundiced neonates as a treatment method, without the use of exchange blood transfusion. The study observed a maximum bilirubin level of 25.4 mg/dl and a lowest level of 11.5 mg/dl.

### Factors associated with neonatal jaundice

The binary logistic regression analysis identified independent predictors of neonatal jaundice, including a low APGAR score of < 7, ABO incompatibility, sepsis, Rh incompatibility, and gestational age of < 37 weeks. However, birth trauma, residence, birth asphyxia, PROM, time of delivery, maternal parity, neonatal sex, and family/sibling history were not significantly associated with neonatal jaundice.

In this study, the presence of Rh incompatibility exhibited variability in neonates with jaundice. Neonates with Rh incompatibility were found to have a 30-fold higher likelihood to be affected by jaundice compared with neonates without Rh incompatibility [adjusted odds ratio (AOR) = 30.40, (95% CI = 2.01, 66.20)]. Similarly, the odds of developing jaundice were approximately 9 times higher among neonates who were born with a long duration of labor compared with those born through normal labor [AOR = 9.03 (95% CI = 1.67, 48.33)]. Newborn babies who were delivered before a gestational age of 37 weeks were 6 times more likely to develop neonatal jaundice when compared with babies born at a gestational age of 37 weeks and more (AOR = 6.03, 95% CI = 1.41, 25.79). Similarly, neonates with a low APGAR score had a sevenfold increased likelihood of developing neonatal jaundice compared with neonates with a normal APGAR score [AOR = 7.34 (95% CI = 1.34, 39.65)].

Neonates diagnosed with sepsis were 10 times more affected by neonatal jaundice compared with those who had no sepsis diagnosis [AOR = 10.13 (95% CI = 2.36, 43.56)]. Neonates with blood type incompatibility were 24 times more likely affected by neonatal jaundice compared with those without blood type incompatibility [AOR = 24.55 (95% CI = 1.58, 68.8)] (Table 3).

**Table 3:** Bivariable and multivariable logistic regression analyses of factors for neonatal jaundice neonates admitted to NICU in Dessie Town public hospitals in the Amhara region, Ethiopia, 2020.

Variable (*N* = 218)	Category	Jaundice		COR [95% CI]	AOR [95% CI]
		Yes	No		
Maternal parity	Prime para	38	34	5.68 [3.05, 10.74]	5.58 [1.66, 18.82]
	Multi para	24	122	1	1
Residence	Urban	28	92	1	1
	Rural	34	64	1.75 [0.96, 3.16]	1.84 [0.99, 3.38]
PROM	Yes	14	9	3.18 [1.36, 7.44]	1.38 [0.22, 8.78]
	No	48	147	1	1
Gestational age	< 37 weeks	48	85	2.86 [1.46, 5.62]	6.03 [1.41, 25.79]*
	> 37 weeks	14	71	1	1
Neonatal sex	Male	41	69	2.46 [1.33, 4.55]	5.42 [1.37, 21.49]
	Female	21	87	1	1
Birth asphyxia	Yes	8	4	5.63 [1.63, 19.45]	2.40 [0.30, 19.60]
	No	54	152	1	1
Sepsis	Yes	24	31	2.55 [1.34, 4.85]	10.13 [2.36, 43.56)*
	No	38	125	1	1
Prolonged labor	Yes	12	9	3.52 [1.38, 8.99]	9.03 [1.67, 48.33]*
	No	50	147	1	1
Birth trauma	Yes	22	9	8.98 [3.84, 21.03]	22.65 [3.64, 74.82]
	No	40	147	1	1
Family/sibling history	Yes	19	7	10.83 [4.06, 28.81]	13.01 [4.79, 35.38
	No	43	149	1	1
ABO incompatibility	Yes	12	7	4.84 [1.36, 17.16]	24.55 [1.58, 68.83)*
	No	47	152	1	1
Low APGAR score	Less than 7	42	66	2.80 [1.52, 7.03]	7.34 [1.34, 39.65]*
	More than 7	20	90	1	1
Rh incompatibility	Yes	7	5	4.47 [1.04, 19.33]	30.40 [2.01, 66.20]*
	No	55	151	1	1

*P-value <0.05 with 95% CI.

## Discussion

This study revealed that the proportion of neonatal jaundice was found to be 62 cases (28.4%). This finding was consistent with studies conducted in India and Pakistan (11, 19). However, it was lower than the prevalence found in cross-sectional studies conducted in Addis Ababa, Southeast Nigeria, Nigeria, Gondar, sub-Saharan Africa, and northern Ethiopia (13, 17, 20–23). This may be due to differences in the study area, study design, time variability, and methodology. The multivariable logistic regression analysis revealed that a low APGAR score of < 7, ABO incompatibility, sepsis, prolonged labor, Rh incompatibility, and gestational age < 37 weeks were found to be independent predictors of neonatal jaundice.

Neonates who had ABO incompatibility were 24 times more likely to be affected by jaundice compared with those without ABO incompatibility. This finding was supported by the studies conducted in Nigeria (24). Neonates who had sepsis were approximately 10 times more affected by neonatal jaundice compared with those who had no sepsis diagnosis. This is in line with the studies conducted in Nigeria (11) and South India (25) and the study conducted by Israel-Aina and Omoigberale (26). This might be because hemolysis, hepatocellular damage, ileus, and/or acidosis may occur as a result of sepsis. These factors may increase bilirubin production (hemolysis), decrease bilirubin removal (liver cell damage), and increase the reabsorption of bilirubin or hepatic dysfunction, resulting in the accumulation of serum bilirubin in the body (23, 27). Neonates with preterm birth were six times more likely to be affected by neonatal jaundice compared with neonates with a gestation period of 37 weeks or more. This result is in line with the studies conducted by Narayan in India and Nigeria (17, 21). Infants who were delivered prematurely (less than 37 weeks) were at a higher risk to have jaundice due to the immaturity of their bilirubin conjugating system, a higher rate of hemolysis, increased enterohepatic circulation, and decreased caloric intake (28). Another neonatal variable that was found to be significantly associated with neonatal jaundice was a low APGAR score of < 7. Specifically, neonates with a low APGAR score of < 7 were approximately eight times more likely to be affected by neonatal jaundice compared with those who had an APGAR score of > 7. This finding was supported by the studies conducted in Nigeria [(25) South India (16), which revealed that a low APGAR score below 7 is an independent risk factor for neonatal jaundice. The APGAR score is the overall indicator for of the newborn’s condition in the extra uterine environment, and neonates with low APGAR scores could be in a state of bradycardia asphyxia and sepsis, which can potentially lead to neonatal jaundice (29). The odds of jaundice were about nine times higher among neonates who were born with a long duration of labor compared with those born in normal labor. This finding was in line with the findings in other studies (23, 30). This might be attributed to the occurrence of bruising and swelling on the scalp of newborns due to the excessive pressure exerted by birth attendants as a solution for prolonged labor, which in turn, increases the risk of jaundice by increasing bilirubin levels in the blood. Neonates with Rh incompatibility were 30 times more affected by neonatal jaundice compared with the newborn without Rh incompatibility. This finding was supported by a study conducted in West India (18). This might be due to the fact that the mother is Rh-negative and the fetus is Rh-positive, and some fetal RBCs cross the placenta and enter the maternal circulation through a minor tear or at the time of delivery; fetal red cells sensitize the mother to antigens on the surface of the fetal red cells, causing the synthesis of anti-D, IgM, and IgG antibodies, leading to neonatal jaundice (22).

## Strength of the study

Various data collection tools were employed to gather information from diverse sources and enhance the credibility of the study. The data collection instrument underwent a preliminary evaluation, while the principal investigator supervised the entire data collection procedure.

## Limitations of the study

This study utilized a cross-sectional study design, which examines the exposure and outcome simultaneously. However, it does not establish causality and relies on previously collected secondary data that may be subject to change.

## Conclusion

The findings of this study suggest that neonatal jaundice was prevalent at Dessie Town public hospitals, with a prevalence rate of 62 (28.4%) cases. Factors such as low Apgar score, prematurity, blood type incompatibility, prolonged labor duration, and neonatal sepsis were found to be significantly associated with neonatal jaundice. In order to prevent and detect neonatal jaundice early, it is essential to conduct blood group testing for all women as part of their antenatal follow-up, particularly those with blood type O. In addition, healthcare professionals should strictly adhere to aseptic techniques while performing invasive procedures on newborns, and policymakers should strive to meet the overall satisfaction of women's needs. By implementing these measures, we may effectively address the issue of neonatal jaundice and improve the health outcomes for both mothers and infants.

## Data Availability

The raw data supporting the conclusions of this article will be made available by the authors, without undue reservation.
